# The functional cooperation of 5-HT_1A_ and mGlu4R in HEK-293 cell line

**DOI:** 10.1007/s43440-020-00114-1

**Published:** 2020-05-29

**Authors:** Grzegorz Burnat, Piotr Brański, Joanna Solich, Magdalena Kolasa, Barbara Chruścicka, Marta Dziedzicka-Wasylewska, Andrzej Pilc

**Affiliations:** 1 Department of Neurobiology, Maj Institute of Pharmacology, Polish Academy of Sciences, Smętna Street 12, 31-343 Kraków, Poland; 2 Department of Pharmacology, Maj Institute of Pharmacology, Polish Academy of Sciences, Smętna Street 12, 31-343 Kraków, Poland; 3 Drug Management Department, Faculty of Health Sciences, Institute of Public Health, Jagiellonian University Collegium Medicum, Grzegorzecka 20, 31-531 Kraków, Poland

**Keywords:** Cell signaling, Gpcrs, Cross-talk, mGlu4 receptors, 5-HT1A receptors

## Abstract

**Background:**

The serotonin 5-HT_1A_ receptor (5-HT_1A_R) and metabotropic glutamate receptor 4 (mGlu4) have been implicated as sites of antipsychotic drug action. 5-HT_1A_R belongs to the A class of G protein-coupled receptors (GPCRs); mGlu4 is a representative of class C GPCRs. Both receptors preferentially couple with Gi protein to inhibit cAMP formation. The present work aimed to examine the possibility of mGlu4 and 5-HT_1A_ receptor cross-talk, the phenomenon that could serve as a molecular basis of the interaction of these receptor ligands observed in behavioral studies.

**Methods:**

First, in vitro studies were performed to examine the pharmacological modulation of interaction of the mGlu4 and 5-HT_1A_ receptors in the T-REx 293 cell line using SNAP- or HALO–tag and cAMP accumulation assay. Next, the colocalization of these two receptors was examined in some regions of the mouse brain by applying RNAScope dual fluorescence in situ hybridization, immunohistochemical labeling, and proximity ligation assay (PLA).

**Results:**

The ex vivo and in vitro results obtained in the present work suggest the existence of interactions between mGlu4 and 5-HT_1A_ receptors. The changes were observed in cAMP accumulation assay and were dependent on expression and activation of mGlu4R in T-REx 293cell line. Moreover, the existence of spots with proximity expression of both receptors were showed by PLA, immunofluorescence labeling and RNAscope methods.

**Conclusion:**

The existence of interactions between mGlu4 and 5-HT1A receptors may represent another signaling pathway involved in the development and treatment psychiatric disorders such as schizophrenia or depression.

**Electronic supplementary material:**

The online version of this article (10.1007/s43440-020-00114-1) contains supplementary material, which is available to authorized users.

## Introduction

The G protein-coupled receptors (GPCRs) form the largest and most diverse receptor superfamily in mammals [1]. The International Human Genome Sequencing Consortium reported a total of 569 rhodopsin-like GPCRs [2]. It has been estimated that approximately 34–50% of all modern drugs act on GPCRs [3].

Data suggesting the possibility of GPCR oligomer formation were reported for the first time in the 1970s; the formation of β-adrenergic receptor homodimers was described [4]. Then, in the early 1990s, Ferre and coworkers [5] demonstrated GPCR heteromer formation between adenosine A2 and dopamine D2 receptors in rat striatal membranes. Thus, it has been postulated that oligomerization might provide an additional level of signaling diversity and complicity provided by cross-talk between two different receptors. The interaction of receptors like cross-talk or oligomerization might induce functional and structural changes that lead to new features of established protein complexes, affecting binding sites, modulating GPCR signaling and activating alternative signaling pathways.

In the present study, we examined the possibility of interaction of members of two different classes of GPCR. First, the metabotropic glutamate 4 receptor (mGlu4) is a representative of class C GPCRs [6, 7]. It is a large protein of approximately 105 kDa assembled from 912 amino acids (aa). mGlu4 shares structural features common to mGlu receptors with large extracellular domains, seven transmembrane domains and a C-terminal tail. The extracellular part consists of a venous flytrap domain (VFT) with a binding site for orthosteric ligands and a cysteine-rich domain linked with 7TM. Like other members of group III mGlu receptors, its activation inhibits the activity of adenylate cyclase (AC), thus decreasing cAMP concentration in cells [8]. In the central nervous system (CNS), the mGlu4 receptor is predominantly presynaptically expressed within the active zone of neurotransmitter release and controls synaptic firing. The highest expression of mGlu4 is observed in the cerebellum as well as in the cerebral cortex and the hippocampus [9]. The preclinical data show that orthosteric agonists and positive allosteric modulators (PAMs) of mGlu4 receptors induce a dose-dependent reversal in the positive, negative and cognitive symptoms observed in an animal model of schizophrenia [10–12].

The 5-HT_1A_ receptor belongs to the A class of GPCRs and consists of 421 aa in humans. Its N-terminus (extracellular part) consists of no more than 30 aa. Similar to the mGlu4 receptor, the serotonin receptor 1A preferentially couples with Gi/o protein to inhibit cAMP formation [13]. In the brain, presynaptic and postsynaptic pools of 5-HT_1A_ receptors exist. The presynaptic 5-HT_1A_ receptors are located in the dorsal raphe and function as somatodendritic autoreceptors on serotoninergic nerve endings; the postsynaptic receptors can be found in the CA1 area and the dentate gyrus of the hippocampus [14]. Moreover, the 5-HT_1A_ receptor activates phosphatidylinositol-specific phospholipase C and other phospholipases, activates several different protein kinases and regulates the function of several distinct types of ion channels [13]. The 5-HT_1A_ receptor was considered for many years to be a target for the treatment of a number of CNS disorders, including depression [15] and schizophrenia.[16].

Both receptors have been shown to interact with other GPCRs. For example, 5-HT_1A_ can interact with serotonin 5-HT_7_, dopamine D2 and μ-opioid receptors [17, 18].The interaction of mGlu4 receptor with other mGlu receptors from groups II and III (but not group I) [19] has been shown. To date, there is no information concerning the interaction between 5-HT_1A_ and mGlu4 receptors. However, the existing data suggest that the antipsychotic-like activity of mGlu4 orthosteric agonists and PAMs were dependent on serotonergic signaling via 5-HT_1A_ receptors [20] because the effects of sub effective doses of mGlu4 agonists/PAMs in several behavioral tests were potentiated by sub effective doses of the selective 5-HT_1A_ agonist (R)-(+)-8-hydroxy-DPAT [21], while the antipsychotic-like activity of the effective doses of the mGlu4 compounds was blocked by the selective 5-HT1_A_ receptor antagonist WAY100635 [22].

Therefore, the present work aimed to examine the possibility of mGlu4 and 5-HT1_A_ receptors cross-talk—the phenomenon that could serve as a molecular basis of the interaction of these receptor ligands observed in behavioral studies. In vitro studies were performed to examine the pharmacological modulation of the mGlu4 and 5-HT1_A_ receptor signaling in the T-REx 293 cell line. We evaluated the pharmacological response of mGlu4 and 5-HT_1A_ receptors by measuring cAMP levels upon stimulation with receptor agonists, L-Glu, (R)-(+)-8-OH DPAT or both. Moreover, the cells could be considered as the endogenous source of the glutamate which can stimulate mGlu4R and influence the activity of the test system. Thus, the effect of an inhibitor of the glutamate antiporter, the sulfasalazine (SSZ) was investigated. Additionally, potential molecular interaction was measured by a HTRF-FRET assay in the presence of L-Glu, (R)-(+)-8-OH DPAT. To examine whether the interaction between 5-HT_1A_ and mGlu4 exists in the natural neural system, colocalization of the receptors was examined in chosen regions of the mouse brain using proximity ligation assay (PLA).

## Materials and methods

### Plasmid constructs

A DNA fragment encoding variant 1 of human metabotropic glutamate receptor 4 (*GRM4*, NM_000841) was obtained from the University of Missouri, and the fragment encoding serotonin receptor 1a (*5-HT*_*1A*_) was obtained from Origene. The SNAP-tag and Halo-tag plasmids ware obtained from New England Biotech.

The *GRM4* sequence was subcloned into the pcDNA5/FRT/TO multi cloning site as described by Chruścicka et al. [23]. Then, after the 28th amino acid of the signaling peptide sequence containing the site for AgeI and SbfI, restriction enzymes was inserted using the QuikChange Lightning Site-Directed Mutagenesis Kit (Aligent Stratagene) according to the manufacturer’s instructions. Two primers were used to introduce the restriction sites: CCTTCCTCCCTGGGAACCGGTTTCCCTGCAGGAAAGCCCAAAGGCC and GGCCTTTGGGCTTTCCTGCA GGGAAACCGGTTCCCAGGGAGGAAGG. Thereafter, sequencing (primer: AGGCTTGGTGATGATGGGTG) and restriction analysis was performed to confirm the introduction of the new restriction sites (Fig. S1). A fragment encoding the SNAP protein was subcloned from the pSNAP vector into a modified sequence of *GRM4* by AgfI and SbfI enzymes. Insertion was confirmed by restriction analysis, immunostaining and functional assay. *5-HT1A* was subcloned from the pcDNA3.1 plasmid into the pCLIP-Vector (BamHi and XhoI). Due to the lack of satisfactory substrate specificity between the SNAP-tag and CLIP-tag, the CLIP sequence was changed to the HALO-tag. The pHTN HALO-Tag CMV-neo vector (Promega) fragment containing the HALO sequence and part of the CMV promoter was exchanged with a similar sequence in the CLIP-*5-HT1A* plasmid (NdeI and SbfI).

The T-REx 293 cell line (Invitrogen) is recombinant HEK-293 cell line transfected with tetracycline-inducible gene expression system. The cell line was maintained in DMEM medium supplemented with 10% FBS (tetracycline free), 2 mM Glutamax I (Lonza,), 100 µg/mL Zeocin and 10 µg/mL blasticidin (Invitrogen). T-REx 293 cells were treated with a mixture of plasmids (0.1 µg pTet-SNAP-*GRM4* and 0.9 µg pOG44) and GeneJuice transfection reagent according to the manufacturer’s instructions (Novagen). After 48 h, selection for the stably integrated plasmid with 100 µg/mL hygromycin B (Invitrogen) began. In parallel experiments, the HALO-*5-HT*_*1A*_ plasmid was introduced into T-REx 293 cells or cells with the inducible expression of SNAP-*GRM4* to generate a cell line that expressed both receptors. In the double expression system (as well as in other experiments), 5-HT_1A_ was stably expressed, and mGlu4R expression was induced by tetracycline treatment (+ Tet; 0.75 µg/mL) (Fig. S3). One additional cell line was generated by cloning the *5-HT*_*1A*_ insert (BamHI/XhoI) into the pSNAPf vector (New England Biotechnologies).

### Forskolin-induced cAMP accumulation assay

The determination of intracellular cAMP using a homogeneous time-resolved fluorescence (HTRF) cAMP dynamic 2 kit from Cisbio was performed according to our previously described methodology [23]. Briefly, cells were grown in DMEM medium with FBS and without tetracycline. Forty-eight hours before experiments, mGlu4 receptor expression was induced by adding 0.75 µg/mL tetracycline. 24 h before the experiment, FBS was removed, cells were scraped and centrifuged. The cell pellet was suspended in Hanks-HEPES buffer (130 mM NaCl, 5.4 mM KCl, 1.8 mM CaCl_2_, 0.8 mM MgSO_4_, 0.9 mM NaH_2_PO_4_, 20 mM HEPES, and 3.25 mM glucose; pH 7.4) and it was incubated in the presence of 5 µM forskolin and the following agonists: l-glutamate; (R)-(+)-8-OH DPAT; WAY100635, a 5-HT_1A_ receptor antagonist; and VU0155041, a positive modulator of mGlu4 receptors. After 10 min incubation, 10 µL cell suspension was incubated with 5 µL cAMP-d2 conjugate and 5 µL anti-cAMP cryptate conjugate. Following 1 h of incubation at room temperature), the fluorescence at 620 nm and 665 nm was determined (Tecan Infinite M1000). The results were calculated as the 665 nm/620 nm ratio multiplied by 10^4^. Additionally, the effect of sulfasalazine, an inhibitor of the cystine-glutamate antiporter on cAMP level in T-REx 293 expressing both receptors was examined. SSZ was present in culture medium without L-Glu from the time point of tetracycline addition and over cAMP assay in concentration 100 µM.

### In vitro receptor interaction assays

T-REx 293 cells expressing one or both labeled receptors were harvested from the cell culture flask, centrifuged and suspended in PBS at 2 × 10^6^ cells/mL (without Ca^+2^/Mg^+2^, with 3.25 mM glucose and 0.1% BSA). Thereafter, the cells were incubated with the following fluorescence substrates for 2 h at 37 °C with slow agitation (90 rpm on an orbital shaker): 100 nM Snap-Tb (Cisbio), 150 nM Snap-Green (Cisbio) and 200 nM Halo-Alexa 488 (Promega) then washed and centrifuged three times to remove unbound substrates. In the final step, the cells were suspended in Hanks-HEPES buffer and fluorescence signals were measured on a TECAN Infinity M1000 (white 384 low volume plate; 20,000 cells per well in a total volume of 20 μL; Greiner BIO-ONE) in two channels: (1) donor Snap-Tb (ex. 320 nm; em. 490 nm; delay 50 μs, integration 700 μs) and (2) FRET signal (ex.320 nm; em. 520 nm; delay 50 μs, integration 700 μs). The intermolecular interaction was presented as a ratio (FRET/Snap-Tb) x10e^4^. Receptor interactions were analyzed in the presence of the mGlu4 receptor agonist l-glutamate; the 5-HT_1A_ agonist (R)-(+)-8-OH DPAT [24]; the positive modulator of mGlu4 receptors VU0155041 [25]; and the 5-HT_1A_ receptor antagonist WAY100635 [26].

### Animals

Animal studies were carried out in accordance with the European Community guidelines and national law. The mouse C57BL/6J (the Jackson Laboratory) in the age of three mount had free access to food and water and were kept at constant room temperature (24 °C), under 12-h light/dark cycle. After decapitation, mouse brains were removed, immediately frozen on dry ice and stored at − 80 °C until cryosectioning.

### RNAscope in situ hybridization assay

The RNAscope in situ hybridization (ISH) procedure described by Wang et al. was applied [27]. Coronal sections (thickness: 10 μm) were cut with a Cryostat (Leica Microsystems GmbH, CM 3000) at − 20 °C and thaw-mounted directly onto Super Frost Plus slides (Menzel). The slides were stored at − 20 °C until ISH processing.

RNAScope ISH for Htr1a and GRM4 mRNAs was manually performed according to the User Manual for Fresh Frozen Tissue using the RNAscope Multiplex Fluorescent Reagent Kit (Advanced Cell Diagnostics Srl, Italy). The slides with brain sections were fixed in 4% paraformaldehyde in PBS. The target probes for specific mRNAs (Htr1a and GRM4) were applied to the brain sections and incubated at 40 °C for 2 h in the EZ Hybridization Oven. These probes were as follows: GRM4-C1 probe (accession number (NM_001291045.1; target nucleotide region: 1076-2146); Htr1a-C2 probe (accession number NM_008308.4; target nucleotide region: 563–1824). Finally, the sections were incubated for 30 s with 4′,6-diamidino-2-phenylindole (DAPI) to stain nuclei (blue) and the slides were mounted with ProLong Gold Antifade Mountant (Thermo Fisher Scientific).

Fluorescent images were acquired using the Axio Imager 2 fluorescence microscope equipped with excitation and emission filters compatible with the fluorophores used, 20 × and 40 × objectives, and a camera (Carl Zeiss MicroImaging GmbH, Germany).

### Double immunofluorescence labeling

Brain coronal sections (thickness: 10 µm) were fixed in 4% paraformaldehyde in PBS for 10 min and rinsed in PBS (3 times for 5 min each) incubated in blocking solution (5% normal donkey serum in PBS) for 30 min at RT, then overnight at 4 °C with a mixture of primary antibodies. Final concentration of anti-5-HT1A receptor antibody (goat polyclonal, ab101914, Abcam) was 1:200, and of the anti-mGlu4 receptor antibody (rabbit polyclonal, SAB4501322, Sigma Aldrich) it was 1:50. On the following day, the sections were incubated for 1 h at room temperature with secondary antibodies: Alexa Fluor 488 donkey anti-rabbit IgG and Alexa Fluor 555 donkey anti-goat IgG (Thermo Fisher Scientific), then washed 3 times with PBS and mounted using DAPI-containing mounting medium (Sigma Aldrich). Images were acquired using an Axio Imager 2 fluorescence microscope (Carl Zeiss MicroImaging GmbH, Germany) equipped with excitation and emission filters compatible with the fluorophores.

### In situ proximity ligation assay

Receptor–receptor interactions were estimated using the Duolink II in situ Proximity Ligation Assay (PLA) detection kit (Sigma Aldrich) according to the manufacturer’s protocol. Slides with tissue sections (thickness: 5 µm) were fixed in 4% paraformaldehyde in PBS (Sigma Aldrich). and incubated overnight at 4 °C in Duolink II Antibody Diluent solution with a mixture of primary antibodies: rabbit polyclonal anti-mGlu4 receptor antibody (1:100, SAB4501322, Sigma Aldrich) and goat polyclonal anti-5-HT1A receptor antibody (1:200, ab101914, Abcam) fallowed by incubated with PLA probes (Duolink II anti-Goat-MINUS and Duolink II anti-Rabbit-PLUS) for 1 h at 37 °C in a humidified chamber.. Then, the ligation reaction was performed at 37 °C for 30 min in a humidified chamber. Amplification mixture was added for 100 min at 37 °C in a darkened humidified chamber. Immunofluorescence staining and positive PLA signals were detected using the Axio Imager 2 fluorescence microscope with Zen 2 Pro Software (Carl Zeiss MicroImaging GmbH, Germany).

### Data analysis

Data obtained from cAMP assay and FRET experiments were analyzed with Prism Version 5.04 (GraphPad Software Inc.). Each experiment was performed three times (*n* = 3), and each data point was in triplicate. The curves for the data shown were fitted to a three-parameter logistic equation, allowing for the determination of IC_50_ values and *E*_max_. For comparison of IC_50_ between the curves, *t* test was applied. The ONE way ANOVA and Tukey’s post hoc test were used for in vitro receptor interaction assays statistics.

## Results

### cAMP accumulation assays

In all experiments, cells expressing tagged receptors as for FRET measurement were used.

**Fig. 1:**
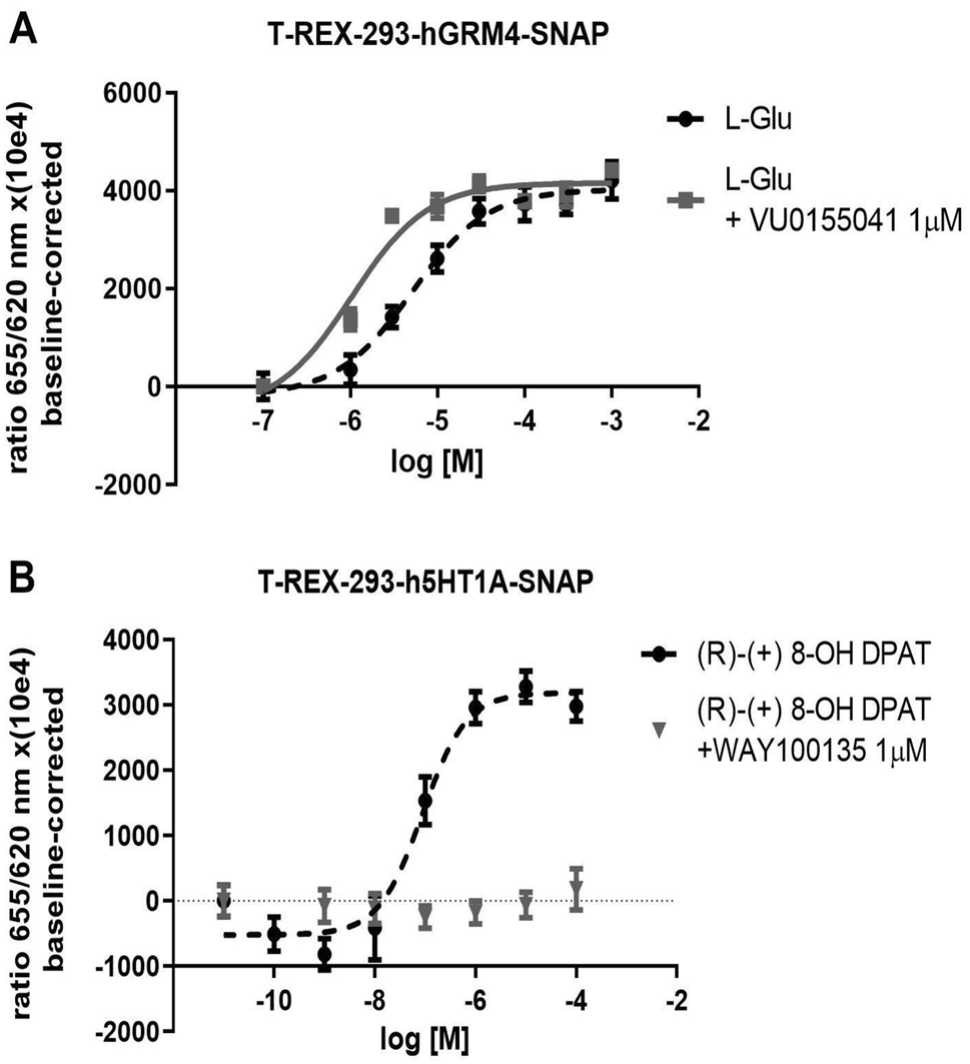
**a** The curve of representative effects of increased concentrations of L-Glu on cAMP accumulation with or without the presence of VU0155041, an mGlu4 receptor PAM in cells overexpressing human GRM4 fused with SNAP. The results show mean ± SD from three replicates. **b** The 5-HT1A agonist (R)-(+)-8-OH DPAT induced inhibition of 5 µM forskolin-stimulated cAMP production in T-REx 293 cells overexpressing human 5HT_1A_ receptor tagged with Snap protein. The effect of the agonist was abolished by the addition of an 5-HT1A antagonist WAY100135

The IC_50_ of the inhibition of forskolin-stimulated cAMP production by l-glutamate in cells expressing mGlu4 receptors (T-REx-293-GRM4-SNAP cells) was 15.14 μM [± 5.63 μM (SD)] (Fig. 1A). The positive allosteric modulator of mGlu4 receptors VU0155041 (1 µM) shifted the curve to the left, decreasing the IC_50_ value to 3.74 μM [± 4.03 μM (SD)] (*t* test: *p* = 0.048; *t* = 2.837). Average shift of IC_50_ value for PAM was 3.98 [± 1.89 (SD)].

The IC_50_ for the inhibition of forskolin-stimulated cAMP production by an agonist of 5-HT_1A_ receptors (R)-(+)-8-OH DPAT in cells expressing 5-HT_1A_ receptors (T-REx-293-h5-HT1A-SNAP cells) was 310 nM [± 230 nM (SD)]. The effect of the agonist *E*_max_ was totally abolished by an antagonist of 5-HT_1A_ receptors WAY100135 (Fig. 1b).

**Fig. 2:**
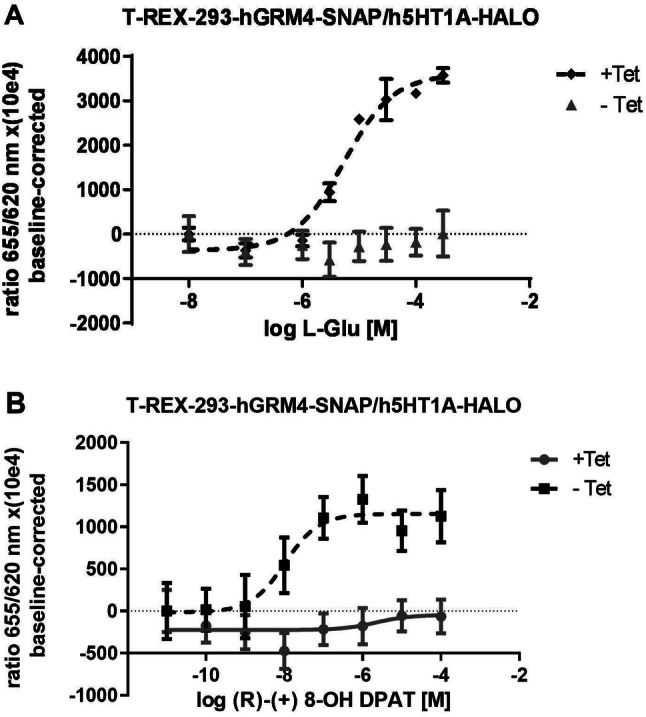
Effect of the coexpression of 5-HT_1A_ and mGlu4 in the T-REx 293 cell line. The expression of mGlu4R was induced by tetracycline administration (+ Tet). **a** Effect of L-glutamate on forskolin-stimulated cAMP accumulation in cells with or without tetracycline induction. **b** Effect of (R)-(+)-8-OH DAPT on forskolin-stimulated cAMP accumulation in cells with or without tetracycline induction

Similar procedure was applied in cells expressing both receptors: 5-HT_1A_-HALO and mGlu4R-SNAP (Fig. 2). After inducing mGlu4R expression, l-glutamate inhibited forskolin-induced cAMP accumulation in a concentration-dependent manner, with an IC_50_ of 5.79 µM [± 3.19 µM (SD)], without the induction of receptor expression, L-glutamate was not effective and determination of the curve parameters was impossible (Fig. 2a). The 5-HT_1A_ receptor agonist action was observed only in cells without tetracycline-induced mGlu4 receptor expression. (R)-(+)-8-OH DPAT without the presence of the mGlu4 receptor (−Tet) displayed an IC_50_ of 62 nM [± 45 nM (SD)] (Fig. 2b). After the induction of mGlu4 receptor expression (+Tet), the effect of (R)-(+)-8-OH DPAT was abolished (Fig. 2b).

**Fig. 3:**
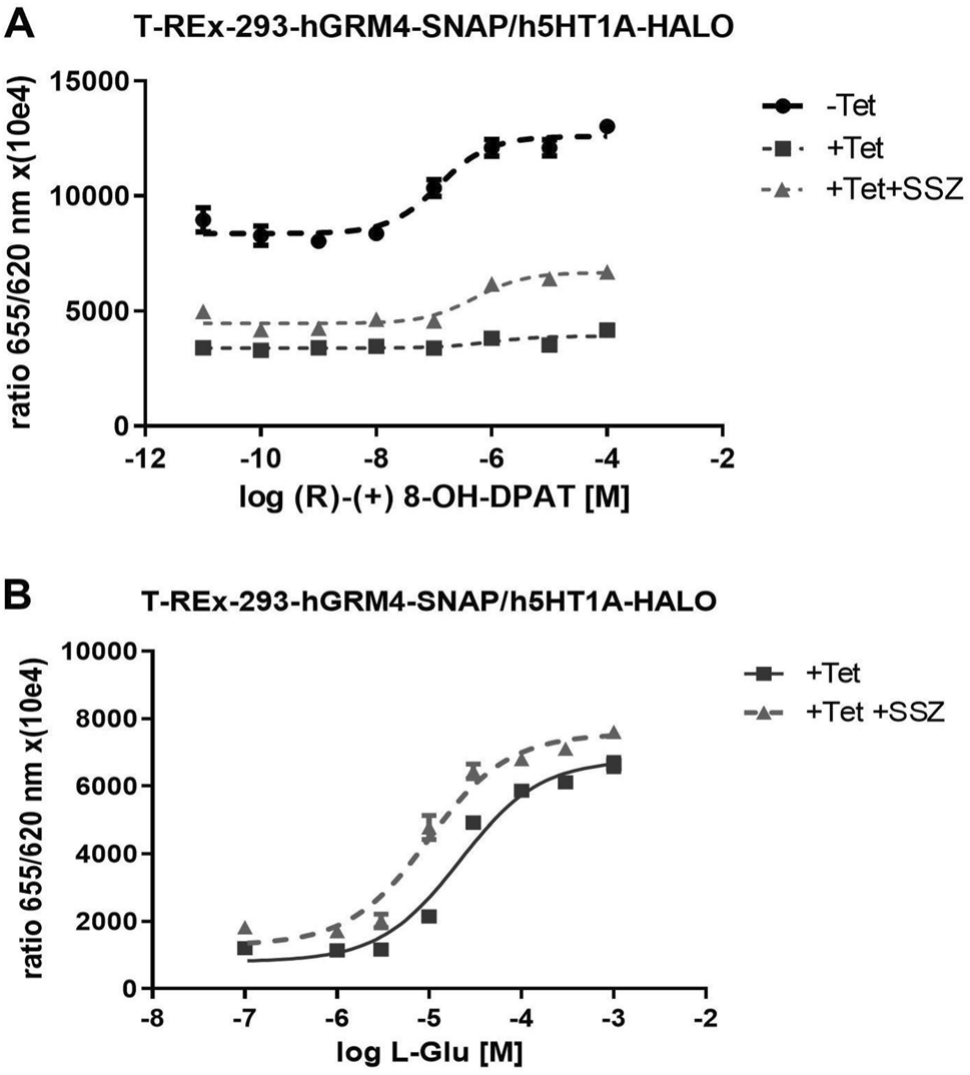
Effect of sulfasalazine (SSZ) at a concentration of 100 μM on receptors activity in cAMP accumulation assay. **a** Sulfasalazine treatment significantly enhanced 5-HT_1A_ activity in presence of mGlu4 receptors (filled triangle) in comparison to cells without sulfasalazine administration (filed square) where expression of mGluR4 induced by tetracycline inhibited the serotonin receptor function. **b** Sulfasalazine treatment shifted the dose–response curve of glutamate to the left

Addition of sulfasalazine (SSZ) to cell culture medium markedly changed 5-HT_1A_ receptor activity in cells cultures coexpressing mGlu4 receptors (+Tet)( Fig. 3). Not only it was possible to evaluate the IC_50_ for 5-HT_1A_ receptor agonist which was 0.30 µM [± 0.24 µM (SD)] but what is important, the E_max_ was 2–3 times higher than in cells without SSZ (Fig. 3a). Concerning the mGlu4 receptors, the IC_50_ for l-glutamate, was decreased in presence of SSZ to 9.78 µM [± 0.99 µM (SD)] from 16.92 µM [± 3.84 µM (SD)] (Fig. 3b) (*t* test: *p* = 0.0356; *t* = 3.119 *df* = 4).

### In vitro receptors interaction assay

At first, the homodimerization of 5-HT_1A_ or mGlu4 receptors fused with SNAP-tag protein and separately expressed in the T-REx 293 cell line was analyzed. A homogenous time-resolved FRET assay was used to analyze the interaction between receptors labeled with Snap-Tb and Snap-green. For each experiment, a group named “mix cells” (1:1) was added, which corresponded to a mixture of cells separately labeled with one substrate depending on the experimental procedure and mixed immediately before FRET measurement.

**Fig. 4:**
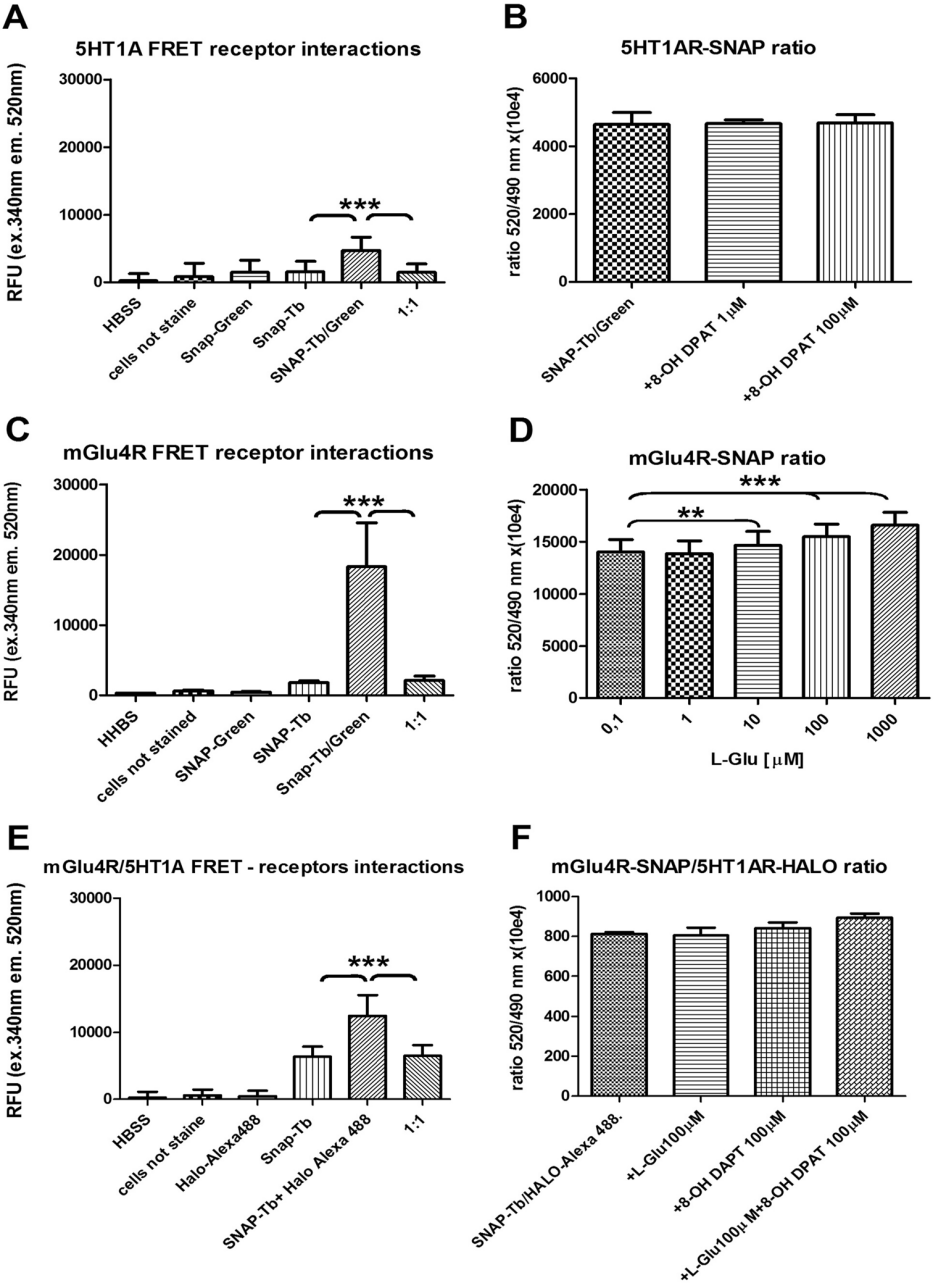
FRET intensity in T-REx 293 overexpressing system between the receptors labeled with Snap-Tb (donor) and Snap-Green (acceptor) presented on the surface of the cells (**a**, **b** 5-HT_1A_; receptor cells, **c**, **d** mGlu4 receptor cells). The signal was measured at the specific emission of 520 nm for the acceptor and at 490 nm for the donor after excitation at 340 nm. **a** and **c** FRET intensity in single- and **b** and **d** in double-labeled cells. The ratio of FRET and Snap-Tb × 104 in double-labeled cells treated with agonist (R)-(+)-8-OH DPAT (**b**) or l-glutamate (**d**). **e** and **f** Demonstrate the interaction between mGlu4 and 5-HT_1A_ receptors tagged with Snap-Tb and Halo-Alexa 488, respectively, presented on the surface of the T-REx 293 cell line. **e** A significant (*p* < 0.001) increase in FRET fluorescence was observed in the double-labeled cells. **f** The effect of glutamate and/or (R)-(+)-8-OH DPAT on the ratio of FRET and Snap-Tb/Halo-Alexa 488 × 10^4^. ONE-way ANOVA **p < 0.01, ***p < 0.001

The 5-HT_1A_ receptor showed a significant increase in FRET signal in double-labeled cells compared to the buffer alone, nonlabeled cells, single-labeled cells, or cell mixture alone (1:1), which indicates homodimerization of the 5-HT_1A_ receptor (Fig. 4a). In all experiments, the increase varied from 1.6 to 3.3. The ratio of FRET to Snap-Tb signal (520/490), which corresponded to interactions between receptors labeled with both fluorescence probes, did not show any changes in the presence of two concentrations (1 or 100 μM) of the 5-HT_1A_ receptor agonist, (R)-(+)-8-OH DPAT (Fig. 4b).

The mGlu4 receptor showed significant increase (× 60 times) in FRET signal in double-labeled cells as compared to nonlabeled cells, buffer or cell mixture alone, suggesting the close proximity of the mGlu4 monomers that formed homodimer in the T-REx 293 cell line (Fig. 4c). The endogenous mGlu receptor agonist l-glutamate induced a concentration-dependent increase in the HTRF ratio 520/490 (Fig. 4d).

When the interaction between mGlu4 and 5-HT_1A_ receptors tagged with the Snap-Tb and Halo-Alexa 488 substrates, respectively, was analyzed, the FRET signal of the cells labeled with both substrates was significantly higher in comparison to the single substrate-labeled cells or cell mixtures, what indicated that these two receptors form heterodimers (Fig. 4e). However, the calculated 520/490 nm ratio in cells incubated with (R)-(+)-8-OH DPAT (100 μM) or L-Glu (100 μM) did not change (Fig. 4f). These results indicate that the proximity of both receptors expressed in the T-REx 293 cell line was not changed in the presence of agonists.

**Fig. 5:**
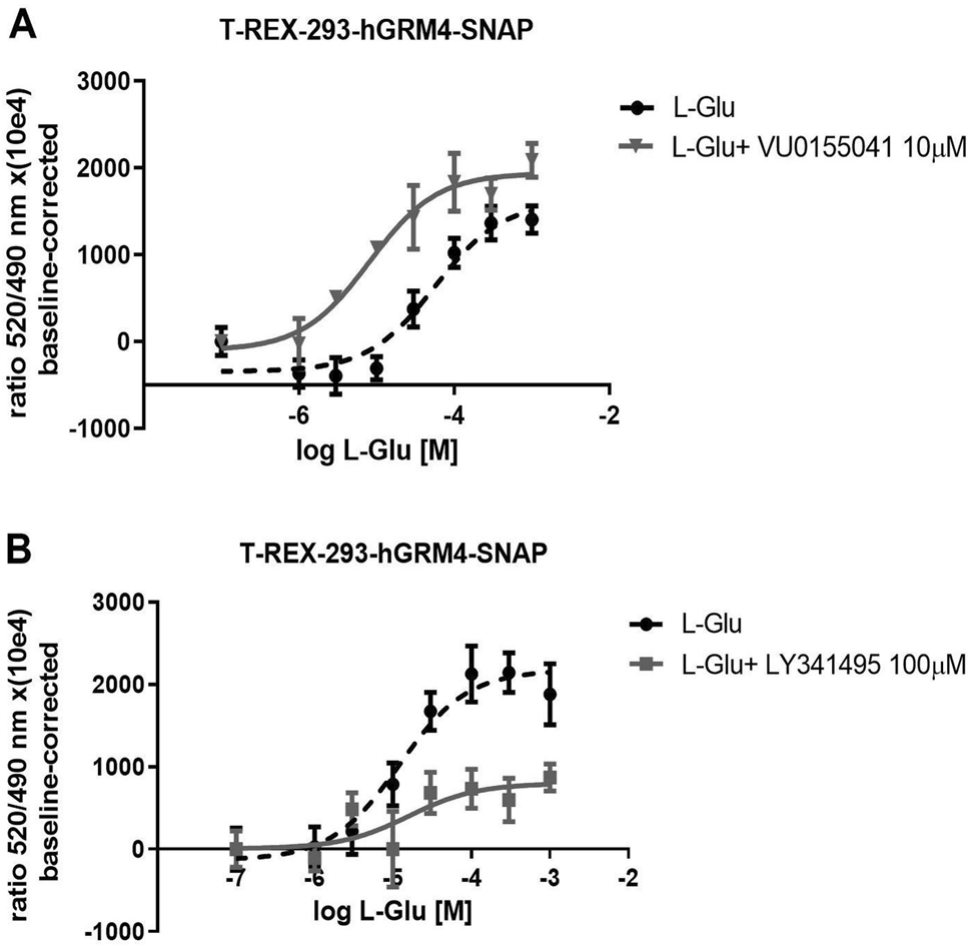
Representative FRET results demonstrating changes in the interaction between the hmGlu4 receptor on the surface of T-REx 293 cells. To label receptors on the cell surface, we used SNA-Tb and SNAP-green membrane-impermeable substrates. **a** The effect of L-glutamate alone or in the presence of VU0155041 (10 μM). In the presence of VU0155041, the positive allosteric modulator, this process was enhanced (*t* test *p* < 0.0001 *t* = 18.0236 *df* = 4). **b** The effect of L-glutamate alone or in the presence of LY341495 a nonselective antagonist, which decreased the FRET to SNAP-Tb signal ratio. LY341495 in concentration 100 µM exerted strong antagonistic effects, shifting the concentration curve to the left and downwards. For L-glutamate IC_50_ was 48.18 μM, SD ± 16.15 μM

The influence of positive allosteric modulator (PAM) VU0155041 and non-selective antagonist of group II/III mGlu receptors LY341495 on FRET signal are presented on Fig. 5. PAM enhanced interaction between mGluR4 monomers labeled with donor or acceptor. Shift of IC_50_ for PAM was 5.11 [± 0.84 (SD)]. An opposite effect was observed upon administration of the antagonist of group II/III mGlu receptors LY341495, which significantly attenuated the FRET signal between the mGluR4 monomers. Shift of IC50 for NAM was 3.98[± 0.73 (SD)].

### In situ mGlu4 and 5-HT1A receptor expression

**Fig. 6:**
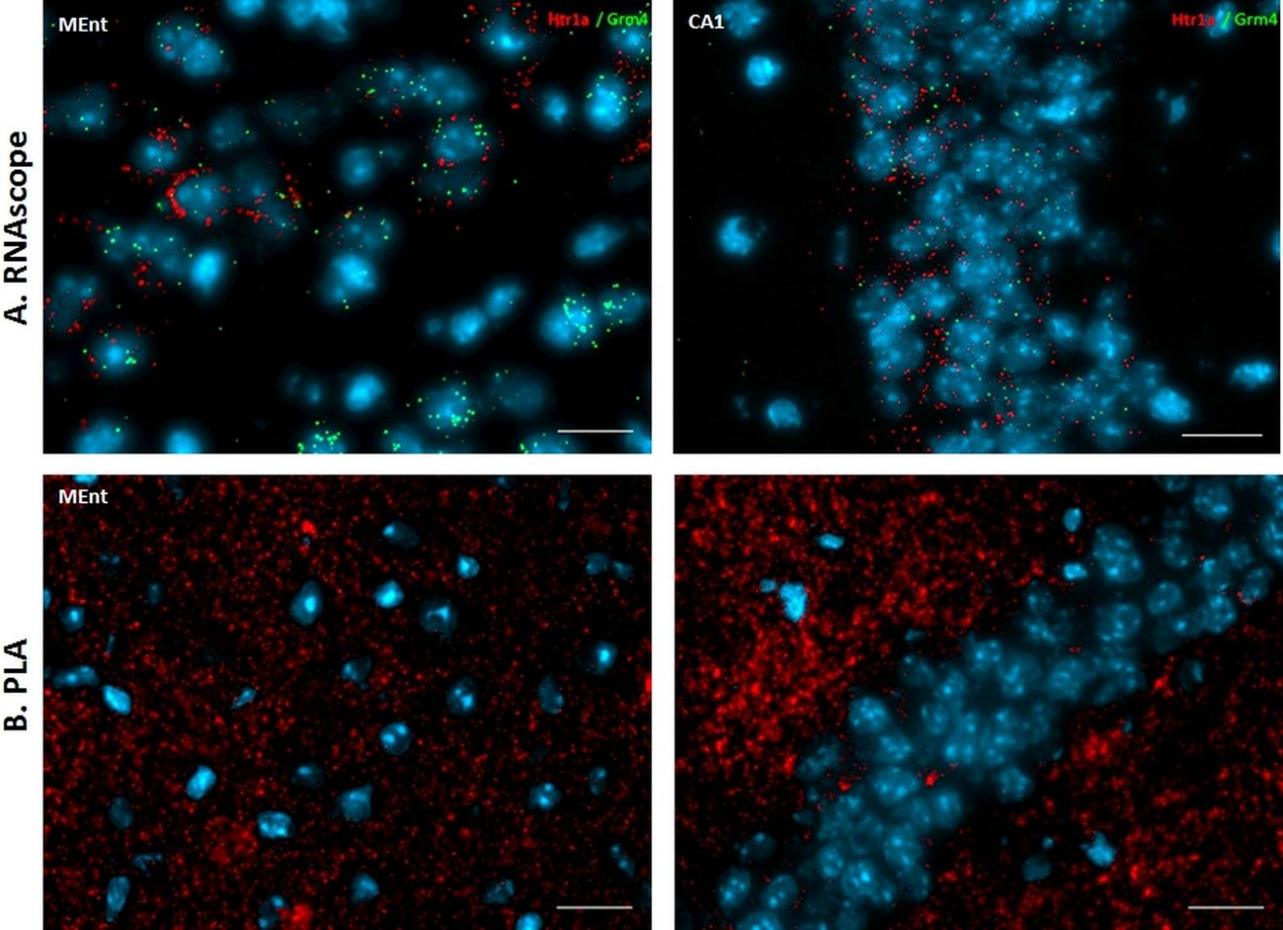
**a** Dual RNAscope for Htr1a (red) and GRM4 (green) probes in the medial entorhinal cortex (MEnt) and the CA1 field of the hippocampus (CA1). Nuclei are stained with DAPI (blue). **b** mGlu4/5-HT_1A_ receptor heteromerization in the mouse brain mGlu4/5-HT_1A_ receptor heteromerization was visualized by in situ PLA. The brain regions for PLA analysis were chosen according to Paxinos and Franklin (2001). mGlu4/5-HT_1A_ receptor heteromers as red dots in MEnt and CA1. Nuclei are stained blue and scale bars = 20 µm

The expression levels of mRNAs encoding the mGlu4 receptor (GRM4) and the 5-HT_1A_ receptor (Htr1a) were detected using the RNAScope dual fluorescence in situ hybridization method which allows for the visualization of a single molecule of mRNA [27]. The expression of the studied transcripts was evaluated in MEnt and CA1 (Fig. 6a) according to the mouse brain atlas (Paxinos and Watson, 1986). The obtained signal (green dots for GRM4 and red dots for Htr1a) indicated that mRNAs encoding of GRM4 and Htr1a receptors are co-expressed in the enthorinal cortex (MEnt). The abundance of GRM4 and Htr1a mRNA was also observed in some cells in the CA1 field of the hippocampus.

The occurrence of mGlu4-5-HT1A receptor heteroreceptor complexes was studied by PLA method in the CA1 and MEnt. The PLA (red dots), indicating close mGlu4 and 5-HT1A receptor proximity (< 17 nm) was confirmed in both regions of the mouse brain (Fig. 6b).

**Fig. 7:**
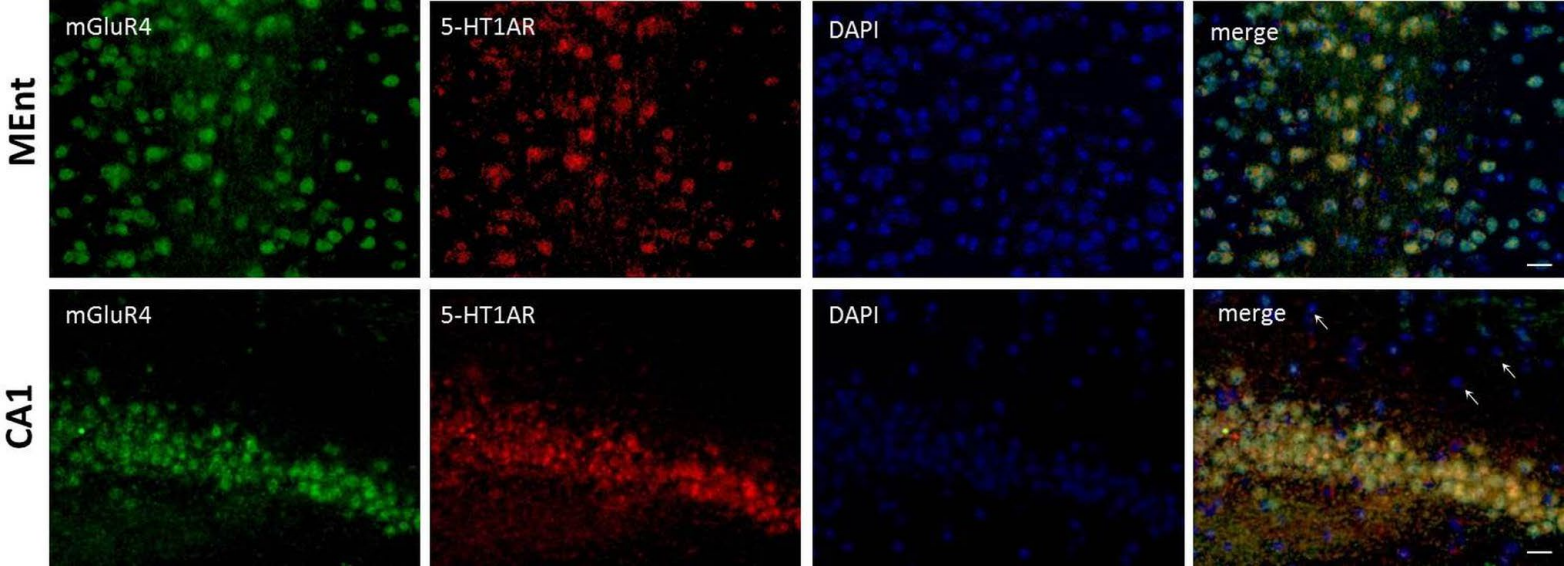
mGlu4 and 5-HT_1A_ receptors co-localize in different regions of the mouse brain. The sections of the brain labeled with anti-mGlu4 receptor primary antibody and detected with Alexa488 donkey anti-rabbit secondary antibody (green) are shown in the first column. The sections labeled with anti-5HT_1A_ receptor (5-HT_1A_R) primary antibody and detected with Alexa555 donkey anti-goat secondary antibody (red) are shown in the second column. The nuclei labeled with DAPI (blue) are shown in the third column. Figure 6 also demonstrated the specificity of antibody anti 5-HT_1a_ and anti-mGluR4 used for colocalization staining. Both demonstrated localization in well known and described region of expression like: CA1 piramidal cells layer. In merge arrows indicate some of cells and regions stained with DAPI only where antibodies did not bind to antigens. Scale bars = 20 µm

The colocalization of the proteins of mGlu4 and 5-HT1A receptors visualized by immunofluorescence labelling was detected in the CA1, as well as MEnt (Fig. 7), again confirming the proximity of both receptors.

## Discussion

In the present work, we decided to investigate the possible molecular interaction of the mGlu_4_ and 5-HT_1A_ receptors in T-REx 293 cells line based on previous result obtained in vivo (see “Introduction”).

Receptors functionality was analyzed in the cAMP accumulation assays in the T-REx 293 cell line overexpressing or not mGluR4 and 5-HT_1A_ tagged with –SNAP or –HALO, respectively. Expression of mRNA was quantified by qRT-PCR (Fig. S2). Level of mGluR4 mRNA was increased over 40 times in cells treated with Tet as compared to non-stimulated cells and ratio HTR1A:GRM4 was comparable 1:1.4 in the cells expressing both transcripts. As receptor agonist, we used l-glutamate an endogenous ligand of all mGlu receptors including mGlu4. It is obviously not a selective ligand, however, in the studied system there are no other mGlu receptors that can respond to L-glutamate and downregulate cAMP level after forskolin stimulation beside overexpressed mGlu4 receptors. There were no significant changes in receptor activity in the cell lines expressing one of the receptors without tags (Fig. S4A/B). In the cell line with stable expression of the serotonin 5-HT_1A_ receptor, an agonist, (R)-(+)-8-OH DPAT, inhibited of cAMP production in a concentration-dependent manner, with the IC_50_ of 0.14 µM (Fig. S4A). In the cell line with inducible expression of mGlu4 receptors (Fig. S4B), L-Glu induced a concentration-dependent inhibition of forskolin-stimulated cAMP accumulation with an IC_50_ of 10 µM. These results confirmed that normal biological activity was preserved despite tag sequence insertion into the open reading frame (ORF). However, in the cell line coexpressing the 5-HT_1A_ and mGlu4 tagged receptors, glutamate induced a concentration-dependent inhibition of cAMP accumulation in cells with inducible expression of mGlu4R, while the inhibitory effect of (R)-(+)-8-OH-DPAT was observed only in cells without tetracycline-induced expression of mGlu4 receptors (Fig. 2). This shows that the induction of mGlu4 receptors abolished the inhibitory action of (R)-(+)-8-OH-DPAT and demonstrates that an inhibitory functional interaction between both receptors takes place. The mechanism of this interaction at present is difficult to explain. The canonical mechanism of action of mGlu4 and 5-HT_1A_ receptors is via Gi protein activation. If both receptors have different affinities for the Gi protein in the T-REx 293 cell line, the competition for the same binding site on the Gi protein might favor one receptor that has a better molecular fit. Some of the results presented in other papers might confirm such a hypothesis [28, 29]. Similar effects were observed when 5-HT_1A_ and 5-HT_7_ receptors were co-expressed. Functionally, heterodimerization decreased the 5-HT_1A_ receptor-mediated activation of Gi protein without affecting 5-HT_7_-receptor-mediated signaling [17]. In another work, [30] the co-expression of mGlu2 and mGlu4 receptors in HEK-293 cells had a significant inhibitory effect on *E*_max_ in the cAMP accumulation assay, especially in the presence of a selective receptor agonist as compared to a single receptor-overexpressing cell line. Another premise for cross-talk between mGlu4 and 5-HT_1A_ receptors in T-REx 293 cell line comes from the experiment with SSZ added to culture medium simultaneously with the induction of mGluR4 expression. As an inhibitor of cysteine-glutamate antiporter it decreases the extracellular level of glutamate. One may suspect that a certain amount of glutamate could be secreted to the culture medium and activate mGluR4 receptor, which in turn can dampen the full activity of 5-HT_1A_ receptor. Indeed, the addition of SSZ improved activity of both receptors but this effect was more pronounced for 5-HT_1A_ since SSZ increased both the efficacy and the affinity of 5-HT_1A_ receptor agonist (R)-(+)-8-OH-DPAT. These results confirm the interaction and modulation of 5-HT_1A_ activity in T-REx 293 heterologous system by mGluR4 at least on signaling cascade level. Moreover, all cAMP experiments confirm the proper expression of the tagged and untagged receptors in the cell membrane of T-REx 293cell line and their full activity.

To analyze the direct interaction of the mGlu4 and 5-HT_1A_ receptors, we used the same tagged cell line as for cAMP accumulation assay. One of the available methods that allow for the analysis of the interactions of two proteins is homogenous time-resolved FRET (HTRF). In our experiment, the receptors were tagged with small enzymatic proteins, SNAP or HALO, that selectively recognize and bind fluorescent substrates, and can serve as fluorescence donors or acceptors for FRET experiments. All fluorescence tags used in FRET assay were cell membrane impermeable and they bound to receptors located on the cell surface. We used SNAP-tag in experiments in which the interaction of homoreceptors was analyzed as control experiments for positive interaction between homomers 5-HT_1A_ or mGluR4.

Overexpression of the serotonin 5-HT_1A_ receptor in the heterologous cell system showed a statistically significant increase in the FRET signal (Fig. 4a) as compared to the controls conditions, which indicates close interaction, namely oligomerization or dimerization of 5-HT_1A_R, and remains in agreement with previously published data [31, 32]. We could not observe any changes in fluorescence signal after 5-HT_1A_ receptor agonist administration (Fig. 4b), while Kobe et al. reported that such agonist decreased FRET signal over a few minutes, however, they studied different compound we used the selective 5-HT_1A_ R agonist (R)-(+)-8-OH DPAT because of the possibility of the presence of other serotonin receptors on the HEK-293 cell line, such as 5-HT_7_ or 5-HT_6_ [31, 33].

Interesting data were obtained in the cells expressing the mGlu4 receptor. We confirmed previous results that indicated mGlu4 receptors can form homodimers or homooligomers [19]. In contrast to the 5-HT_1A_ receptors, the FRET signal in the double-stained (SNAP-Tb/ SNAP-green) SNAP-mGlu4 receptor cell line was much stronger than that of the background and controls (Fig. 4c). In the presence of increasing concentrations of L-Glutamate, the FRET signal was increased Fig. 4d), indicating conformational changes in VFT tagged with the SNAP protein, decreasing the distance between the two different VFT domains. These results are in contrast to most available data concerning mGlu receptor interactions. It has been found that following agonist administration, the relative movement of the ligand-binding domain was decreased in mGlu2 tagged receptors [34]. Moreover, recent data also showed a dose-dependent decrease of inter-subunit FRET following L-Glu administration for the mGlu4 receptor [30]. These divergent results concerning VFT movements might arise from differences in plasmid preparation and final SNAP-tag localization on the surface of the cellular membrane. According to the available literature describing the preparation of the construct the mGlu receptor sequence comes after the sequence of the N-signaling peptide of the mGlu5 receptor, and SNAP-tag [19, 35, 36]. Compared to the construct used in the present work, the SNAP-tag sequence is located within the mGlu4 receptor sequence after the mGluR4 its own signaling peptide. This means that the position of the SNAP-tag differs by approximately 30 aa between constructs. Moreover, the sequence that we used in the present work is human complementary DNA. The differences between human and rat mGlu4 receptor sequences and the different preparation of plasmids may contribute to differences in the observed movement of extracellular domain that was tagged with SNAP. Nevertheless, modifications of the N terminus did not affect the function of mGlu4 receptors in the present work or others [30].

In the present work, we also analyzed the influence of the group II/III mGlu receptor antagonist LY341495 [37] and a PAM of mGlu4 receptors VU0155041 [38]. Despite LY341495 being known as mGlu2/3 receptors antagonist it showed a higher potency on mGlu4R than CPPG a selective group III antagonist (Fig. S4C). In the presence of L-Glutamate, administration of PAM dose-dependently enhanced the intersubunit interaction, while the antagonist resulted in the opposite effect, demonstrating that both compounds had an influence on the relative movement of the VFT domain of the mGlu4 receptor where the SNAP tag was located (Fig. 5). According to the current knowledge, modulators bind to allosteric sites located in 7TM. In the present study, PAM not only changed the activity of the receptor but also affected molecular changes in the extracellular part of the mGlu4 receptor, moving the two VFT domains closer and, therefore, increasing FRET efficiency. Thus, this indicates that the extracellular part of the receptor is also affected by molecular changes in the transmembrane domain.

The T-REx 293 cell line that overexpressed both receptors exhibited a statistically significant increase in FRET signal after staining with SNAP and HALO substrates compared to control samples (Fig. 4e). This result indicates that the two receptors are located in close proximity to one another in our experimental heterologous cell system. The FRET signal was within the range of 5-HT_1A_ receptor interactions and was much weaker than the homo-oligomerization of mGlu4 receptors. The pair of 5-HT_1A_ and mGlu4 receptors shows a large difference in the size of the extracellular domain, which might result in a low FRET signal. In other words, the distance between the donor and acceptor might be much greater than the mGlu4-mGlu4 receptor interaction which impairs the energy transfer. According to information included in the patent by Doumazane et al., the energy transfer between donor and acceptor substrates used in HTRF experiments appears at a distance of ~ 20–55 Å (US8697380B2, 2014). Nevertheless, additional procedures like an expression of both receptors in T-REx 293 cell line at a lower level than used in present work and disruption of potential oligomers by means of TAT-peptides technique are necessary to confirm their oligomerizations at least on non-neuronal cells.

To investigate the possibility of 5-HT_1A_ and mGluR4 interaction in natural biological systems we analyzed the expression of mRNA encoding these receptors in the mouse brain. In comparison to methods where antibodies are used, this approach seems more specific in demonstrating the cells and brain regions where such interaction could take place due to the appearance of both transcripts. The results clearly demonstrated that the medial ethorinal cortex (MEnt) is the region where the expression of both receptors is high, which, together with the observation of a strong PLA signal, confirms the close relative position of both receptors. Also the hippocampus (the CA1) represents the region where both receptor proteins were found and the PLA approach confirmed their close proximity. Regarding the CA1, the levels of mRNA transcripts differed slightly from protein expression, what might result from the different half-lives of the same mRNA molecule in various brain structures. As shown with the PLA approach, both receptors are sufficiently close to each other to interact at the molecular level, i.e., forming oligomers or modulating their signaling pathways, in the hippocampus. Our investigations did not allow us to demonstrate the exact cell type that co-expressed the receptors in the MEnt and CA1. In the hippocampus CA1, the immunofluorescence signal from mGlu4 and 5-HT1A receptors was located mainly in the pyramidal and granular cell layers, respectively. Additionally, the PLA method confirmed the high degree of receptor colocalization in the CA1 field of the hippocampus. It is important to mention that immunofluorescence staining gives a linear signal based on protein expression. Due to the high level of signal enhancement in the PLA, this method provides information about where two proteins are located in relation to one another rather than about the expression level.

Results obtained in the present work demonstrate cross-talk between receptors in HEK-293 heterologous expression system and confirm the close proximity of mGlu4 and 5-HT_1A_ receptors in the mouse brain slices what has been suggested in our previous in vivo study. These preliminary findings may represent another signaling route involved in the development of psychiatric disorders such as schizophrenia or depression as well as provide potential new treatment options [39].

## Electronic supplementary material

Below is the link to the electronic supplementary material.

Supplementary file1 (PDF 869 kb)
