# Supplementary material for: The functional cooperation of 5-HT1A and mGlu4R in HEK-293 cell line
Source: Pharmacol Rep. 2020 May 29;72(5):1358–69. doi: 10.1007/s43440-020-00114-1 (PMC7550284; doi:10.1007/s43440-020-00114-1)
Supplement: Supplementary file 1 — Supplementary file1 (PDF 869 kb) [file 43440_2020_114_MOESM1_ESM.pdf]

Supplementary data

The functional cooperation of 5-HT_{1A} and mGlu4R in HEK-293 cell line

G. Burnat[a]*; P. Brański [a]; J. Solich[b]; M. Kolasa[b]; B. Chruścicka[a]; M. Dziedzicka-Wasylewska[b]; A. Pilc[ac]**

[a] *Maj Institute of Pharmacology, Polish Academy of Sciences, Department of Neurobiology, 31-343 Kraków, Smętna Street 12, Poland*

[b] *Maj Institute of Pharmacology, Polish Academy of Sciences, Department of Pharmacology, 31-343 Kraków, Smętna Street 12, Poland*

[c] *Drug Management Department, Institute of Public Health, Faculty of Health Sciences, Jagiellonian University Collegium Medicum, Grzegorzecka 20, 31-531, Kraków, Poland.*

Fig.S1

Sequencing results after mutagenesis:

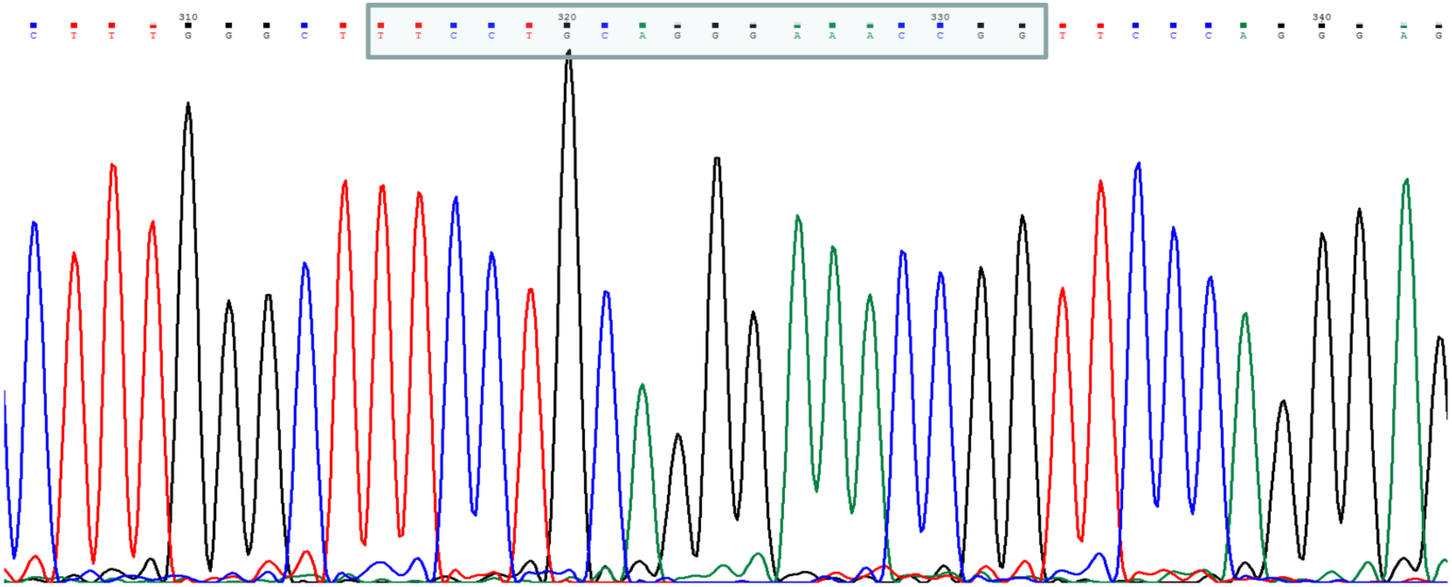

Restriction analysis by EcoRI of pcDNA5-GRM4-SNAP

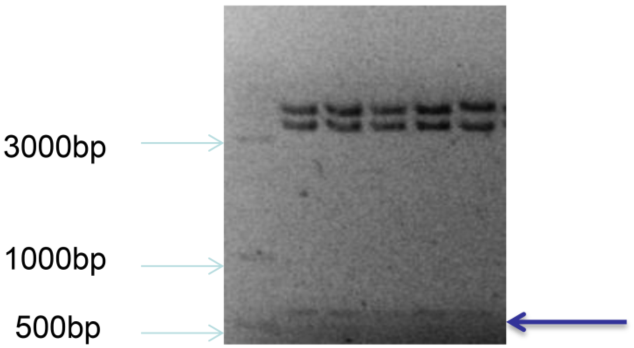

Figure S1. DNA sequencing analysis of pcDNA5-hGRM4 plasmid after site directed mutagenesis and EcoRI restriction analysis of pcDNA5-hGRM4-SNAP

Fig.S2

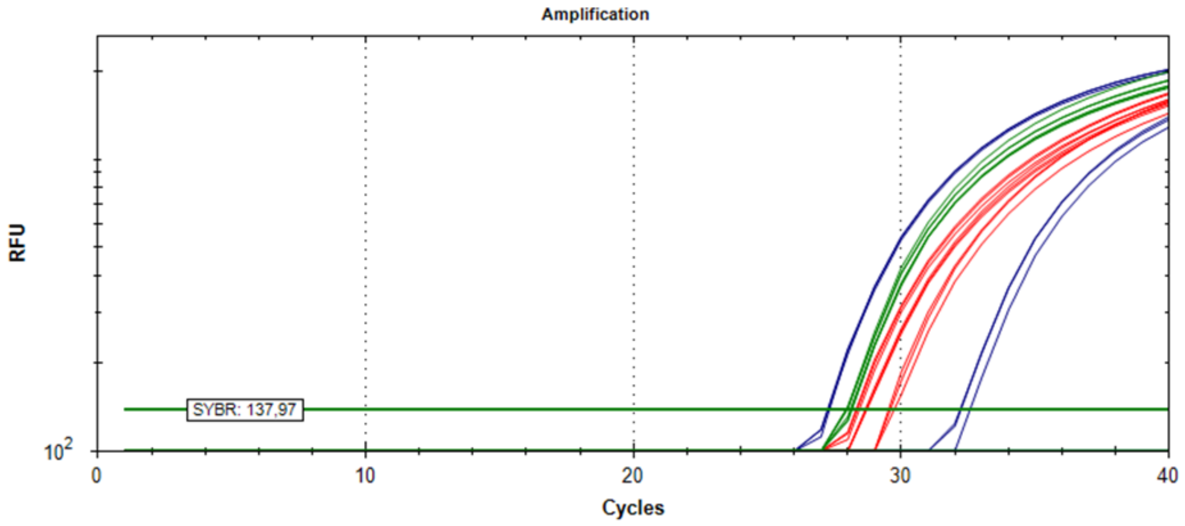

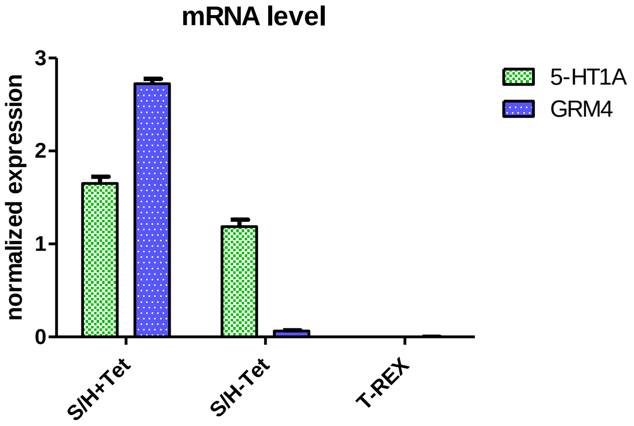

Fig. S2. Compariosn of 5-HT1A and mGluR4 expression in T-REx-293 and T-REx-293-GRM4-SNAP/5-HT1A-HALO cells line with or withot Tet administration. Expression of mGluR4 mRNA was rise over 40 times and ratio 5-HT1A:mGluR4 was 1:1,4 in cells treated with Tet.

Fig.S3

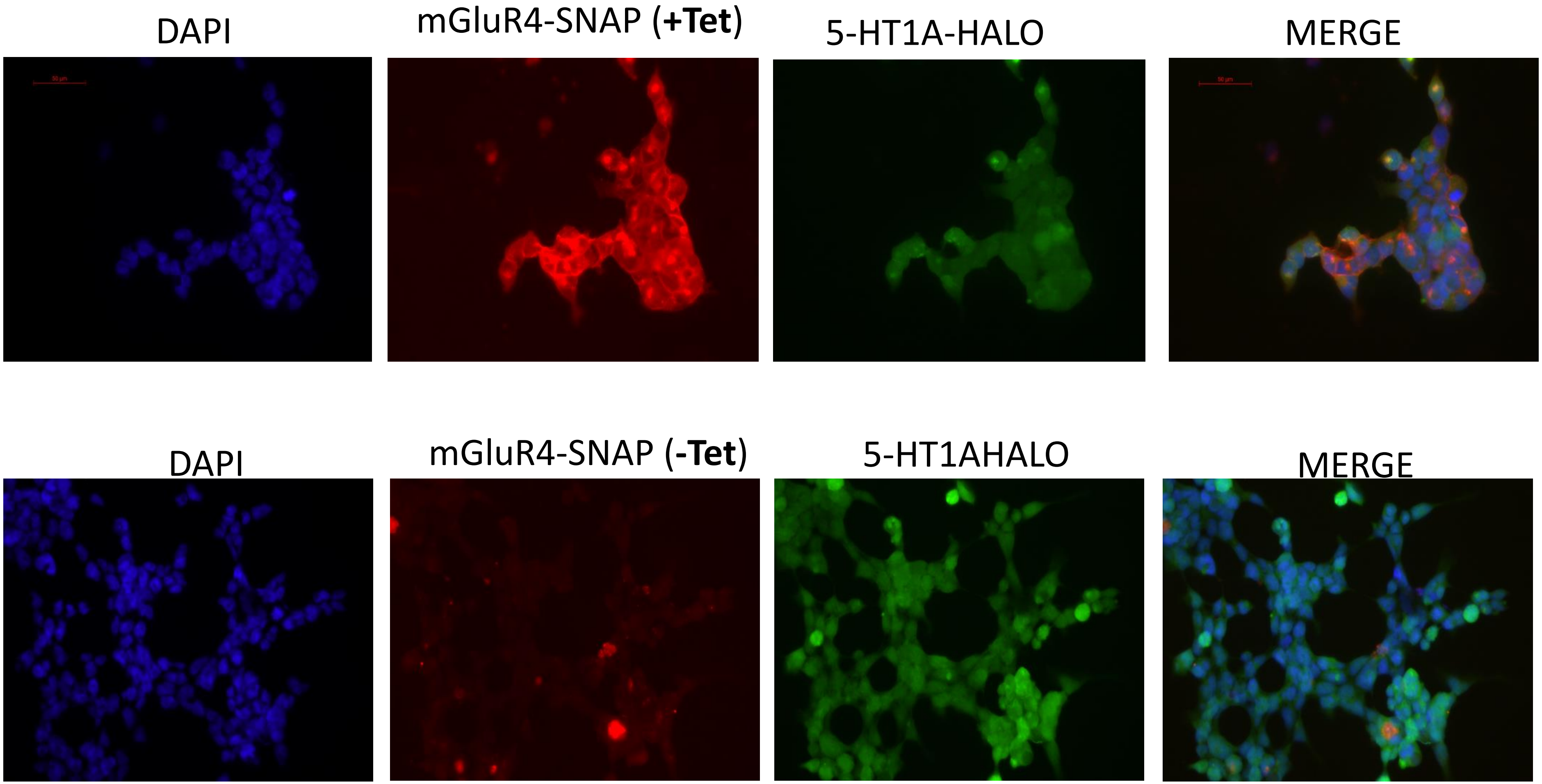

Fig.S3 Fluorescence immgagin of cells expressing taged receptors labeled by Snap-red (Cis-bio) and Halo-Alexa488 (Promega). The cell nuclei was counterstained with DAPI (blue). The Tet treatment turn on expression of mGluR4-SNAP (+Tet) while expression of taged 5-HT1A was stable.

Fig.S4

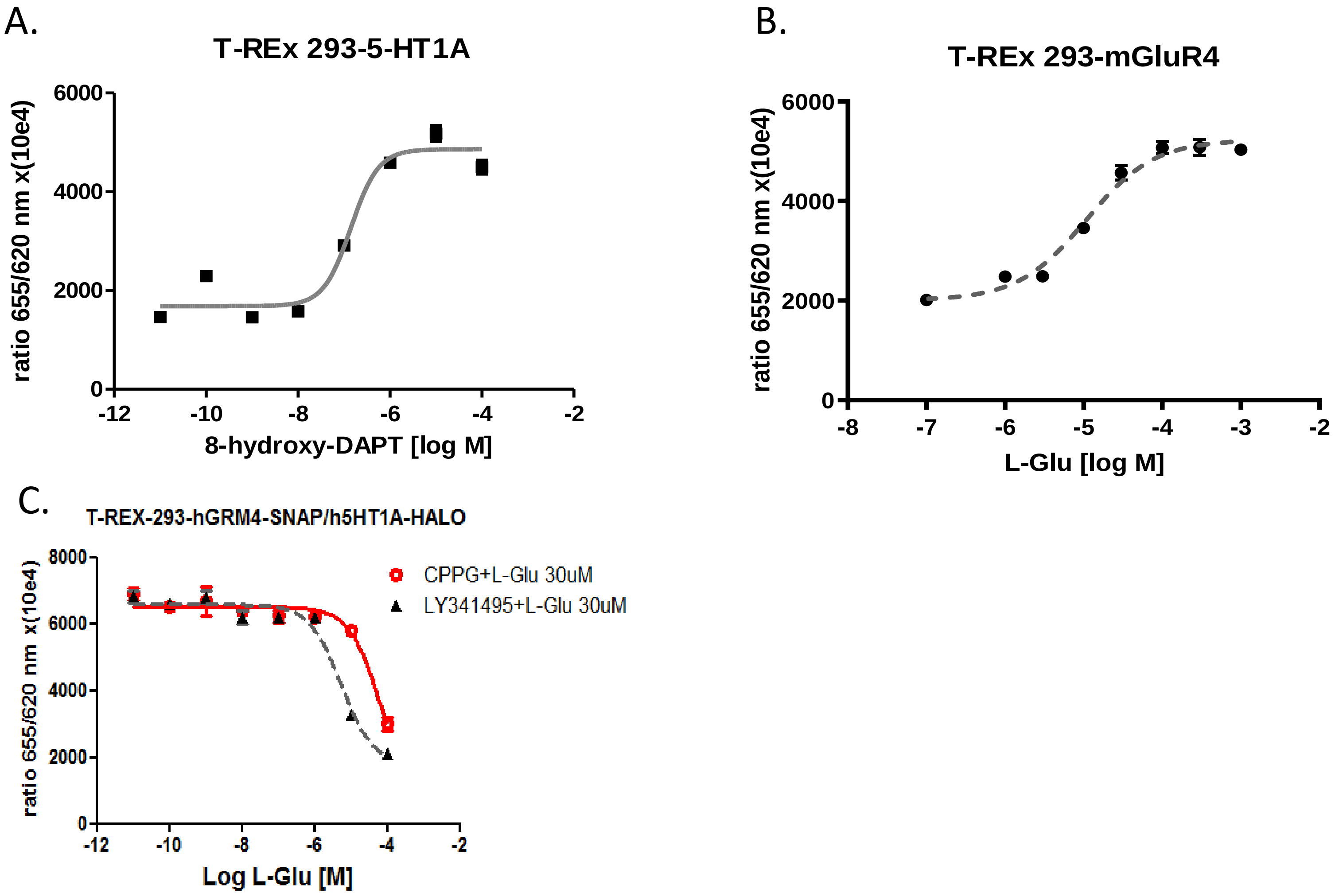

Fig. S4 Representative effects of increased concentrations of **A**): 8-OH-DPAt (0,14 µ M) or **B**): L-Glu (10,76µM) on cAMP accumulation in T-REx 293 cells overexpressing human 5HT1A or GRM4 receptor <u>without tags</u>, respectivly. On graphic **C)** is demonstarated effects of selective and unselective mGluR4 antagonist in presence of L-Glu. LY341945 despite lower selectivity was more potent and efficacious compound, therefore it was used throughout experiments.

Fig.S5

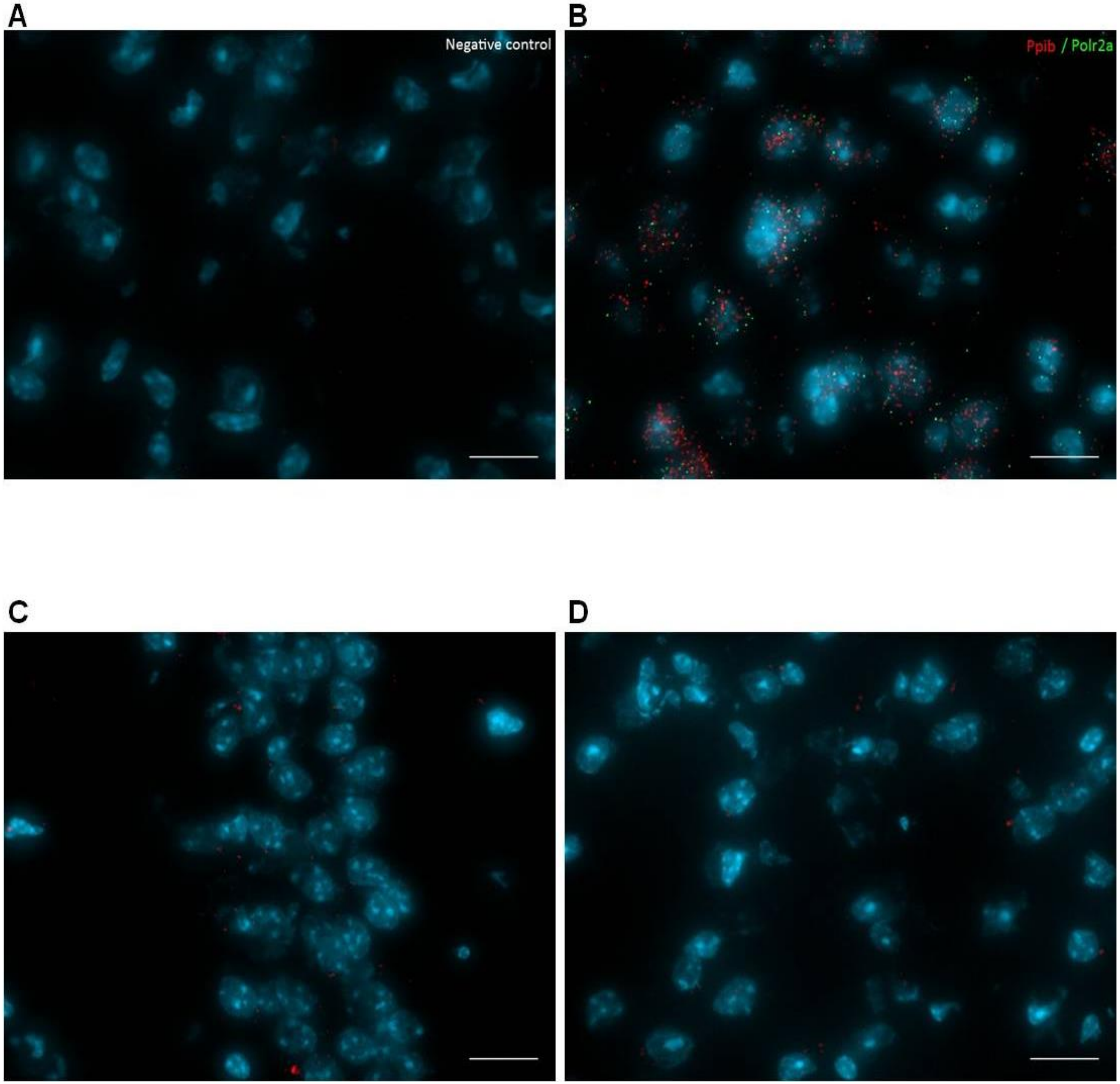

Figure S5. Controls for the RNAscope and PLA methods. No signal in red or green channels was visible in the negative control of the RNAScope method performed with the bacterial DapB probe. Nuclei are stained with DAPI (blue). (B) Positive control dual RNAscope for the Ppib (red) and Polr2a (green) probes was performed in the prefrontal cortex. Nuclei are stained with DAPI (blue). (C) No or negligible signal was visible for the negative control of PLA performed with primary antibodies only for the 5-HT1A receptor. Nuclei are stained blue with DAPI. (D) No or negligible signal was visible for the negative control of PLA performed with primary antibodies only for the mGlu4 receptor. Nuclei are stained blue with DAPI. Scale bars = 20 µm.
